# *In-silico* probing of AML related RUNX1 cancer-associated missense mutations: Predicted relationships to DNA binding and drug interactions

**DOI:** 10.3389/fmolb.2022.981020

**Published:** 2022-08-25

**Authors:** Hanif Ullah, Baoyun Zhang, Narendra Kumar Sharma, Pierre D. McCrea, Yogesh Srivastava

**Affiliations:** ^1^ Guangxi Key Laboratory for Genomics and Personalized Medicine, Guangxi Collaborative Innovation Center for Genomics and Personalized Medicine, Guangxi Medical University, Nanning, China; ^2^ Key Laboratory of Regenerative Biology, South China Institute for Stem Cell Biology and Regenerative Medicine, Guangzhou Institutes of Biomedicine and Health, Chinese Academy of Sciences, Guangzhou, China; ^3^ University of Chinese Academy of Sciences, Beijing, China; ^4^ Department of Bioscience and Biotechnology, Banasthali Vidyapith, Banasthali, Tonk, Rajasthan, India; ^5^ University of Texas MD Anderson Cancer Center UTHealth Graduate School of Biomedical Sciences, Houston, TX, United States; ^6^ Department of Genetics, University of Texas MD Anderson Cancer Center, Houston, TX, United States; ^7^ Genome Regulation Laboratory; Guangdong Provincial Key Laboratory of Stem Cell and Regenerative Medicine, Guangzhou Institutes of Biomedicine and Health, Chinese Academy of Sciences, Guangzhou, China

**Keywords:** RUNX1, docking, molecular modeling, missense mutation, acute myeloid leukemia

## Abstract

The molecular consequences of cancer associated mutations in Acute myeloid leukemia (AML) linked factors are not very well understood. Here, we interrogated the COSMIC database for missense mutations associated with the RUNX1 protein, that is frequently mis-regulated in AML, where we sought to identify recurrently mutated positions at the DNA-interacting interface. Indeed, six of the mutated residues, out of a total 417 residues examined within the DNA binding domain, evidenced reduced DNA association in *in silico* predictions. Further, given the prominence of RUNX1’s compromised function in AML, we asked the question if the mutations themselves might alter RUNX1’s interaction (off-target) with known FDA-approved drug molecules, including three currently used in treating AML. We identified several AML-associated mutations in RUNX1 that were calculated to enhance RUNX1’s interaction with specific drugs. Specifically, we retrieved data from the COSMIC database for cancer-associated mutations of RUNX1 by using R package “data.table” and “ggplot2” modules. In the presence of DNA and/or drug, we used docking scores and energetics of the complexes as tools to evaluate predicted interaction strengths with RUNX1. For example, we performed predictions of drug binding pockets involving Enasidenib, Giltertinib, and Midostaurin (AML associated), as well as ten different published cancer associated drug compounds. Docking of wild type RUNX1 with these 13 different cancer-associated drugs indicates that wild-type RUNX1 has a lower efficiency of binding while RUNX1 mutants R142K, D171N, R174Q, P176H, and R177Q suggested higher affinity of drug association. Literature evidence support our prediction and suggests the mutation R174Q affects RUNX1 DNA binding and could lead to compromised function. We conclude that specific RUNX1 mutations that lessen DNA binding facilitate the binding of a number of tested drug molecules. Further, we propose that molecular modeling and docking studies for RUNX1 in the presence of DNA and/or drugs enables evaluation of the potential impact of RUNX1 cancer associated mutations in AML.

## Introduction

Cancer associated missense mutations have functional roles in lineage plasticity as well as in tumorigenesis (Srivastava et al., 2020). AML is a hematopoietic malignancy caused by various genetic abnormalities in hematopoietic stem cells (HSCs) that provide obstacles to the normal differentiation process (Kumar 2011; Bonifer and Cockerill 2015). RUNX1 is the most highly mutated gene among leukemias and is an important transcription factor in hematological malignancies (Chin et al., 2016; Menter and Tzankov 2019). RUNX1 consists of an N-terminal RUNT domain important for DNA binding and for forming heterodimers (Kagoshima et al., 1993), while its C-terminal region contains a nuclear localization domain and assists in DNA binding regulation (Mikhail et al., 2006). Aberrations in the RUNX1 gene or its partner CBFB have been involved in the pathogenesis of human myeloid leukemias (Speck et al., 1999). Previous studies show that germline and somatic mutations of RUNX1 are observed in many hematological malignancies such as myelodysplastic syndrome (MDS), acute lymphoblastic leukemia (ALL), acute myeloid leukemia (AML), and chronic myelomonocytic leukemia (CMML) (Sood et al., 2017). Complicated mutational patterns in AML have made targeted therapies difficult because of the arising differences in drug responses (Tyner et al., 2018). Much progress has been made in treatments, but the overall rate is still not satisfactory mainly because of drug resistance. Thus, deeper knowledge is needed of the gene mutations and targeted drug responses to further improve treatments (Berardi et al., 1999). Advances in technology such as NGS (Next Generation Sequencing) have improved insight into the underling molecular mechanism of AML, with the initial successful first-generation drug being imatinib, a tyrosine kinase inhibitor (TKI) used to combat CML (Chronic Myelogenous Leukemia). Second-generation TKIs such as ponatinib, nilotinib and dasatinib then became available for CML (DiNardo and Lachowiez 2019). Many drugs were tested on cell lines to evaluate their efficacy, for example, PTK787/ZK 222584, a molecule that inhibits VEGF (Vascular Endothelial Growth Factor) tyrosine kinase activity, was found to exhibit better activity when used with Idarubicin (Barbarroja et al., 2009). Similarly, missense mutations in RUNX1 are found to co-segregate with AML disease. In particular, the mutations R201Q (Uniprot: R174Q) and R166Q (Uniprot: R139Q) are predicted to disrupt DNA binding, and NMR-derived structures show that both have arginine substitutions at residue positions known to be important for DNA association (Berardi et al., 1999; Song et al., 1999). These mutations provide information that assists in predicting the likely outcome of AML (prognosis) as well as in selecting therapies (DiNardo and Lachowiez 2019).

RUNX1 shows allosteric functions, for example, promoting Ets1–DNA binding through DNA-enhancer driven effects upon the DNA (Shiina et al., 2015). That is, RUNX1 activates the ETS1 transcription factor through the TCR enhancer, enabling ETS1 to play a role in lymphoid differentiation, proliferation, apoptosis, embryonic development, and angiogenesis (Dittmer 2003). This RUNX1-ETS model is helpful in addressing how cancer mutations play roles in DNA mediated allosteric functions and RUNX1-DNA binding.

Here, we asked what are the consequences of selected RUNX1 missense mutations in relation to the association of RUNX1 with DNA, and a range of FDA (Food and Drug Administration) approved drugs, including three designed to treat AML. To address these questions, we evaluated the cancer-associated missense mutations of RUNX1 as obtained from the comprehensive Catalogue Of Somatic Mutations In Cancer (COSMIC) database (http://cancer.sanger.ac.uk) (Bamford et al., 2004). In particular, using in silico methods (Supplementary Figure S1), we identified those residues of RUNX1, mutated in AML, that reside at the critical DNA binding interface of RUNX1. Because the function of RUNX1 is often compromised in AML, we asked the question if these mutations might additionally alter RUNX1’s interaction (off-target) with known FDA-approved drug molecules, including three currently used in treating AML. Our major approach was to map and dock a series of FDA-approved drug compounds. We model two mutations that occur at the same site as present in patient samples of RUNX1. Namely, R174Q (COSM24805) and R177Q (COSM24731), that originate from different tumor types, facilitate binding of the AML associated drug Enasidenib. Our study is thus intended to provide a glimpse of potential mechanisms of action of RUNX1 mutations on DNA association, as well as interactions with drug molecules. Our approach may prove useful for designing targeted therapeutics for AML. Our work, together with that of others, suggests that molecular mutation modeling and docking will prove useful for understanding the molecular consequences of RUNX1 cancer associated mutations. We predict that RUNX1 recurrent mutational hotspot sites provide the field with a valuable guide for the design and modeling of more selective and effective drug molecules.

## Materials and methods

### Data mining

Cancer associated mutation data for the RUNX family of transcription factors were retrieved from COSMIC v85 (https://cosmic-blog.sanger.ac.uk/cosmic-release-v85/). We extracted RUNX1 DNA binding domain associated missense mutations to map them on available RUNX1 complex crystal structures. The mutations’ functionalities were checked and taken into account on the basis of functional analysis through use of the hidden Markov model (FATHMM), with scores provided by the COSMIC database. The FATHMM score ranges from 0 to 1. Mutations were classified as scoring below 0.5 score (neutral), scoring above 0.5 and below 0.7 (deleterious), or scoring greater than 0.7 (pathogenic) (https://cancer.sanger.ac.uk/cosmic). We considered mutants within this pathologic (>0.7) range (Supplementary Figure S1) that were supported by the literature, or where there were indications of an interface between RUNX1 and DNA. We used R packages, “ggplot2” (https://ggplot2.tidyverse.org) (Wickham, 2016), and “data.table” (https://cran.r-project.org/web/packages/data.table/vignettes/datatable-intro.html) to process and visualize the RUNX mutational data from the COSMIC dataset. To avoid duplicates, we excluded different isoforms and evaluated only the primary isoform of RUNX1 that is most widely expressed. We used UNIPROT numbering schemes for the RUNX1 DNA binding domains. Sequence logos for human RUNX1 DNA binding domains were prepared with web logo (https://weblogo.berkeley.edu/logo.cgi). Candidate drugs for docking were selected by examining the literature for a notable functional impact upon their targets. The structures of drug compounds were downloaded from the PubChem database (https://pubchem.ncbi.nlm.nih.gov/).

### Structural modelling and mutation mapping

Crystal structures used in this study were downloaded from the protein data bank. We used structures of the RUNX1-ETS complex on the TCR enhancer (PDB ID: 3WU1) (Shiina et al., 2015), PARP1 (PDB ID: 5XSR) (Chen et al., 2018), EGFRTKI (PDB ID: 5EDP) (Hanan et al., 2016), BCR (PDB ID: 2H32) (Bankovich et al., 2007), and IDH (PDB ID: 6NZM) (Hopkins et al., 2019). Cancer missense mutations were generated on various mutation models for RUNX1 with UCSF Chimera version 1.15 (Pettersen et al., 2004) (http://www.rbvi.ucsf.edu/chimera). Newly generated mutation models of RUNX1-ETS on the TCR enhancer complex were subjected to energy minimization using 1,000 steps steepest decent and 500 steps of the conjugate gradient algorithm with the step size 0.002 Å. The AMBER FF14SB force field (Maier et al., 2015) was used for all protein models, and parmBSC0 was used for all DNA elements for energy minimization. Structural cartoons with highlighted mutation residues were prepared with UCSF Chimera.

### Drug docking

Docking programs are critical for visual illustration of protein-drug binding affinity. In the context of finding the targets of drugs and protein engineering, the prediction of molecular interactions between a protein and drugs or small molecules suggests ways to rationalize the selection of amino acids that could be used to design personalized drugs for specific diseases or mutated to promote or disrupt given interactions (Looger et al., 2003). Interestingly, such information is important to predict the binding affinities of drugs/small molecules with proteins, and thus to estimate biological activities or to help in obtaining new molecular lead compounds or drugs (Boehm et al., 2000; Doman et al., 2002; Huang et al., 2006). For docking of drug compounds with RUNX1, we selected 13 drugs, including three associated with AML and 10 associated with other cancers. These are known cancer drugs, but they do not have any published study showing interactions with RUNX1. In this study we performed blind docking using the Swiss Dock web server (Grosdidier et al., 2011), using ranking of CHARMM energies for all biomolecules (Brooks et al., 2009). We analyzed implicit solvation model clusters of RUNX1 and drug compounds (Haberthur and Caflisch 2008) by using ggplot2 and data.table packages in R to make plots for docking energies. The interpretation of docking results and model figures were prepared by UCSF Chimera version 1.15 (Pettersen et al., 2004).

### Electro-statistics and protein—protein interaction analysis

We used the PDB2PQR tool (Dolinsky et al., 2004; Dolinsky et al., 2007) from UCSF Chimera to prepare structures by adding hydrogens, assigning charges and reconstructing missing atoms after applying AMBER force fields (Ponder and Case 2003; Cheatham and Case 2013; Maier et al., 2015) and generating PQR files. These force fields were used for Poisson-Boltzmann calculations and to prepare structures for APBS (Adaptive Poisson-Boltzmann Solver) for electrostatic analysis (Jurrus et al., 2018). We performed surface electrostatic calculations by applying the PDB2PQR and ABPS tools to probe surface electrostatics difference created by point mutations.

### Functional prediction of mutation impacts

We used the SNAP2 tool from the Predict Protein server that provides predictions for functional secondary-structure changes due to single nucleotide polymorphisms (Reva et al., 2011). Predict Protein server (Yachdav et al., 2014) (https://www.predictprotein.org) Provides measures for transmembrane helices, intra-residue contacts, protein-protein, protein-DNA contacts and clashes, solvent accessibility, disorder regions, domain boundaries, cysteine bonds, di-sulphide bridges, and metal binding sites.

## Results

### Selection and distribution of cancer-associated missense mutations

**FIGURE 1:**
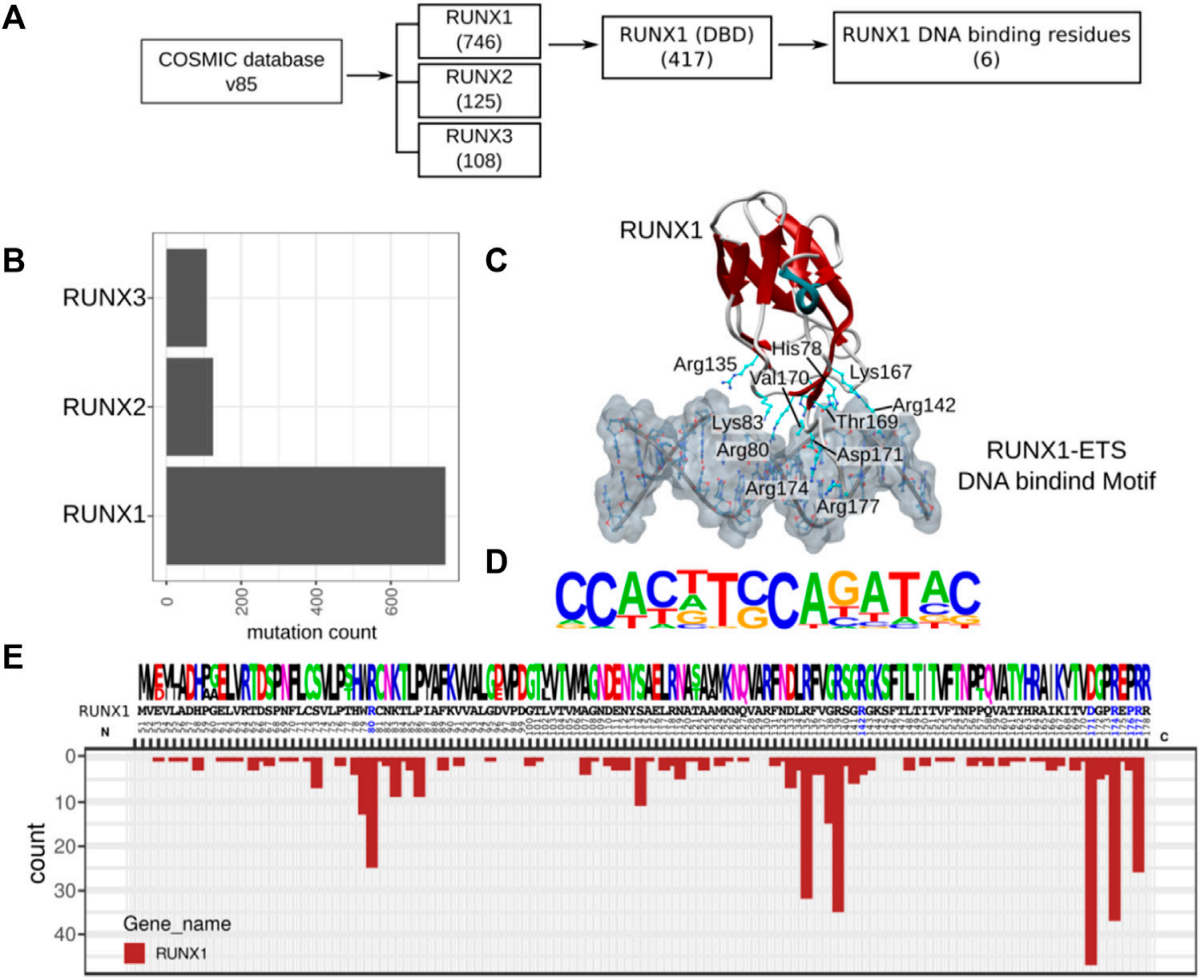
RUNX1 cancer-associated mutation and distribution within its DBD. **(A)** Flow chart for the selection of RUNX1 missense mutations from the COSMIC v85 database for our docking studies **(B)** Cancer-associated missense mutation across the RUNX family. RUNX1 contains approximately 746 missense mutations out of which 417 mutations reside within its DBD. The selection of candidate RUNX1 mutations for docking studies were done based on potential direct DNA binding effects and mutational recurrence. RUNX1 has the highest number of mutations in comparison with RUNX2 and RUNX3. **(C)** Structural cartoon for RUNX1-ETS complex on TCR enhancer (PDB ID: 3WU1) with highlighted DNA-bound six amino acids of RUNX1. **(D)** RUNX1-ETS DNA binding motif from published ChipSeq data (Accession: GSM1527839, GSE17954). **(E)** Conservation of amino acids across the paralogs of RUNX1 followed by distribution of mutations across the DNA binding domain.

We retrieved cancer-associated mutations to identify those with the potential to alter RUNX1: DNA interactions. To this end, we retrieved the COSMIC mutation dataset (release v85) for missense mutations associated with the RUNX family, for which RUNX1, RUNX2, and RUNX3 have 746, 125, and 108 missense mutations, respectively. For RUNX1, out of 746 total mutations, 417 reside within what is defined as the DNA binding domain (DBD). Based upon known RUNX1: DNA (co-crystal) structures (PDB ID: 3WU1) (Shiina et al., 2015), we found a much more restricted set of six mutations likely to have a direct impact upon the DNA association of RUNX1 (Figure 1A). RUNX1 exhibits the highest number of missense mutations in comparison with RUNX2 and RUNX3 (Figure 1B). As noted, we mapped missense mutations to the crystal structure of the RUNX1: DNA complex (PDB ID: 3WU1) to identify interface-interacting amino acid residues within RUNX1. Mutation mapping revealed that positions Arg80, Arg142, Asp171, Arg174, Pro176, and Arg177 interact with DNA, while COSMIC database analysis suggests that these same positions are additionally associated with cancer-associated missense mutations. Although mutations at positions His78, Lys83, Arg135, Thr169, and Val170 are likewise associated with DNA-interactions, they do not appear to be associated with any type of cancer (Figure 1C). To see the DNA binding consequences of cancer-associated missense mutations of RUNX1 and wild type RUNX1, we respectively explored Jurkat-cell RUNX1-ChIP-Seq (GSE17954) and LNCaP-cell RUNX1 Chip-Seq (GSM1527839) (Dasari and Tchounwou 2014), and used the homer motif of the RUNX1-ETS complex for the protein DNA model (Figure 1D). However, with these structural analyses, we did not find visually significant differences for the predicted binding of wildtype versus RUNX1 mutants in binding to DNA. To identify possible drug binding sites, (conceivably providing opportunities for personalized medicine), we inspected the mutation recurrence pattern within the conserved DNA binding domain (DBD) of RUNX1. The amino acid positions Ser73, Trp79, Arg80, Lys83, Pro86, Ser114, Asp133, Arg135, Gly138, Arg139, Gly141, Asp171, Gly172, Arg174, and Arg177 showed highly recurrent mutations while positions 142 and 176, corresponding to an Arg and Pro residue, respectively, have less recurrence in comparison. We also evaluated RUNX2 and RUNX3 for conservation of mutations at the same residue positions as RUNX1 (Figure 1E). Mutations such as at residues Arg80, Asp171, Arg171, Arg177, and Arg142 have been shown present at the protein: DNA contact interface of RUNX1:DNA complex (Bowers et al., 2010). Here, we asked if these mutation positions are likely to actively participate or be relevant to the binding of selected known anti-cancer drugs. To this end we inspected the corresponding protein contact interface of RUNX1 mutants for active drug binding pockets and electrostatics to see the impact of these mutations on drug association.

### Cancer-associated mutations predicted to form active pockets for drug binding

**FIGURE 2:**
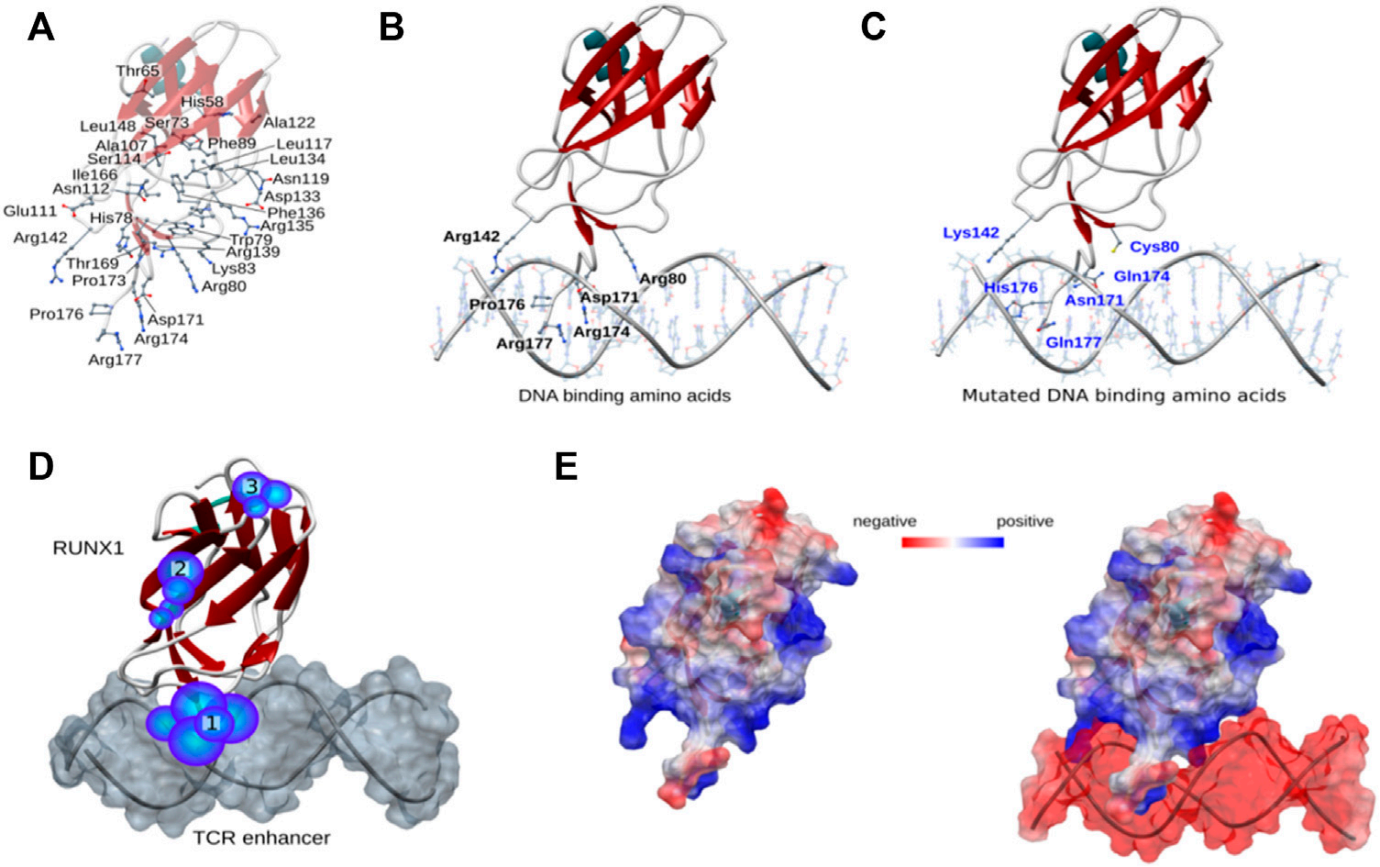
Structural mapping of RUNX1 cancer-associated mutations. **(A)** Mutation sites within the RUNX1 protein structure, showing all 46 recurrent mutations across the DNA binding domain. **(B,C)** Mutation positions associated with DNA binding; wild type amino acids are highlighted with their labels reflecting the uniprot numbering scheme (uniprot ID: Q01196). **(D)** Predicted active pockets for drug binding within the RUNX1-ETS DNA complex. Highlighted numbers show three major pockets for drug binding. **(E)** Electrostatics of RUNX1 and RUNX1 with DNA. Blue indicates highly electropositive surfaces, while red color shows significantly electronegative surfaces for the protein.

We next performed electrostatic analysis for a restricted region within the protein surface of RUNX1 to identify potential active pockets for drug binding. Specifically, we analyzed 46 recurrent cancer associated missense mutation sites for RUNX1 within its DBD (Figure 2A). Out of 46 recurrent positions six sites were located in DBD regions having critical roles in DNA interactions (Figure 2B). Arginine (Arg) makes up 28% of the residues found to interact at the minor groove of DNA only (Rohs et al., 2009). In the case of RUNX1 in the context of its DNA-binding interface, out of the six missense mutations we focused upon, four were Arginine located at positions 80, 142, 174, and 177 that were mutated to Cysteine (Cys), Lysine (Lys), Glutamine (Gln), and Glutamine (Gln), respectively. Thus, the missense mutations under study at the RUNX1: DNA binding interface are Arg80Cys, Arg142Lys, Asp171Asn, Arg174Gln, Pro176His, and Arg177Gln. Of these, we predicted Arg80Cys to be a complete loss-of-contact mutation that could lead to binding disruption of RUNX1 to the T cell receptor (TCR) enhancer (Figure 2C). In contrast, Pro176His is not predicted to alter binding efficiency. Importantly, these mutations excluding Pro176His arose as potentially contributing to forming active binding sites for anti-cancer drugs, based upon predictions of active pockets using the castp web server (Tian et al., 2018). We predicted from our structural analysis that residues Arg80, Asp171, Arg174, and Arg177 may be important positions contributing to active pockets for drug binding to RUNX1. We predicted the potential existence of 23 active pockets from applying the castp web server, and out of these, we evaluated for drug-compound binding at the three active pockets with larger volumes, naming them: pocket 1; pocket2; and pocket 3. Of these, pocket 1 is predicted to be the major pocket given its surface area (Å^2^) (Figure 2D). Delta delta G (ΔΔG) is the predicted binding free energy calculated for drug binding modes and affinities. We used ΔΔG scores as a tool to compare binding affinities of drug compound. Furthermore, electrostatic data for RUNX1 without and with DNA shows that RUNX1 has electropositive areas associated with DNA binding, as well as exposed protein-protein interaction surfaces that exhibit electronegative characteristics, whereas DNA is electronegative in character. The color red is used to depict an electronegative surface while the blue color indicates an electropositive surface (Figure 2E). Importantly, within the RUNX1 active pocket 1 for drug binding, those DNA-binding amino acids that we are focused upon (see above) displayed an electropositive surface. Here, we made predictions based on modeling and docking, that drug binding with RUNX1 inhibits its binding to DNA and thus results in lessened RUNX1 function.

### RUNX1 docking predictions with standard anti-cancer drugs

**FIGURE 3:**
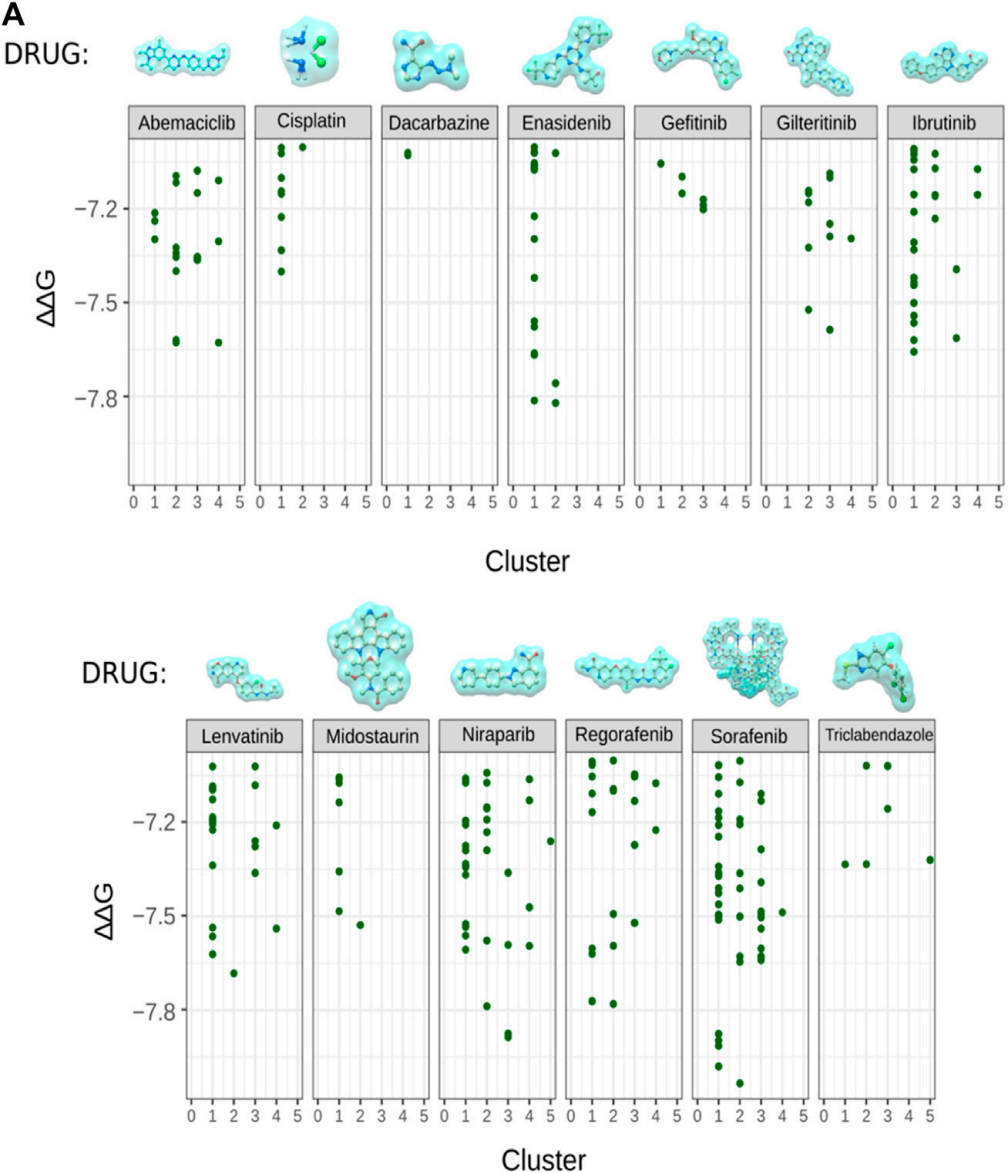
Cancer related drug molecule and their docking with RUNX1. **(A)** Docking of enasidenib (AML), sorafenib (AML), and niraparib (ovarian cancer) with RUNX1-ETS DNA complex displaying the binding position of drug molecules with their ∆∆G values. Showing comparison of docking scores for all 13 drug molecules with RUNX1, we took AML associated drug molecules as positive controls for docking with RUNX1. Sorafenib, regorafenib, niraparib, and enasidenib were indicated by the modeling to have low ∆∆G values suggestive of their drug binding.

In order to predict the stability and strength of interaction of different drugs with RUNX1, an AML drug specific target, we performed molecular docking of standard published Abemaciclib (Gelbert et al., 2014), Cisplatin (Dasari and Tchounwou 2014), Dacarbazine (Serrone et al., 2000) Enasidenib (Del Principe et al., 2019) Gefitinib (Baselga and Averbuch 2000), Gilteritinib (Lee et al., 2017; McMahon and Perl 2019) Ibrutinib (Honigberg et al., 2010) Lenvatinib/Pembrolizumab (Makker et al., 2019), Midostaurin (Manley 2019), Zejula (niraparib), Regorafenib (Carr et al., 2013), Sorafenib (Hotte and Hirte 2002), and Triclabendazole (Fetterer 1986), are employed to treat various cancers—with two being used towards lowering the activity of a corresponding transcription factor in treating AML (Table 1). However, these drugs’ activities and their molecular consequences have not been previously considered in the context of RUNX1-ETS DNA complex. To this end we performed mutation modeling and molecular docking of drug compounds with the RUNX1-ETS DNA complex. Our molecular docking results indicate that with the exception of Dacarbazine, Gefitinib, Cisplatin, and Triclabendazole, the remaining drugs are predicted to have more favorable ΔΔG values when docking wild-type relative to mutant RUNX1. Previous studies have shown that Cisplatin has a number of functional targets including DNA, RNA; sulfur-containing enzymes like metallothionein and glutathione; as well as mitochondria in the case of testicular cancer (Dasari and Tchounwou 2014). Dacrbazine instead leads to the methylation of DNA in the case of melanoma; Gefitinib targets the epidermal growth factor receptor-tyrosine kinase in the case of lung cancer; and Triclabendazole inhibits the binding of 3H-colchicine in the liver fluke *Fasciola hepatica* (Fetterer 1986). A drug compound that is directed towards RUNX1 is Ibrutinib, which affects signaling of the B cell antigen receptor (BCR) in the case of Non-Hodgkin Lymphoma [30]. Sorafenib targets Raf-1 in liver cancer (Wilhelm et al., 2008); Niraparib predominantly binds and inhibits PARP1 and PARP2 in ovarian cancers (From the American Association of Neurological Surgeons et al., 2018); and Enasidenib targets isocitrate dehydrogenase-2 (IDH2) in AML (Del Principe et al., 2019). As the basis for our modeling, we selected drug molecules based on their frequency of use in clinical applications. As cancer drugs often have side effects, we began testing drugs proposed to bind to a diversity of targets. Of the 13 drugs we tested, Enasidenib, Niraparib and Sorafenib exhibited the best-calculated binding (highest ΔΔG) to binding-pocket1 of RUNX1 (Figure 3A). Thus, our docking results predicted that Enasidenib, Niraparib and/or Sorafenib might associate with RUNX1, possibly contributing to under-appreciated off-target effects of these drugs. To this end, we sought to model the binding capabilities of these drugs with wild type RUNX1, versus the above-selected cancer associated missense mutants of RUNX1.

**TABLE 1:** Cancer-associated drug compounds used for docking to RUNX1.

Compound name	Pubchem id	Related cancer
Abemaciclib	46220502	Breast cancer
Cisplatin	5702198	Testicular cancer
Dacarbazine	135398738	Melanoma
Enasidenib	89683805	Acute myeloid leukemia (AML)
Gefitinib	123631	Lung cancer
Gilteritinib	49803313	AML
Ibrutinib	24821094	Non-hodgkin lymphoma
Lenvatinib	9823820	Endometrial cancer
Midostaurin	9829523	AML
Niraparib	24958200	Ovarian cancer
Regorafenib	11167602	Colorectal cancer
Sorafenib	216239	Liver cancer
Triclabendazole	50248	Liver cancer

### Cancer drug associated targets and RUNX1

**FIGURE 4:**
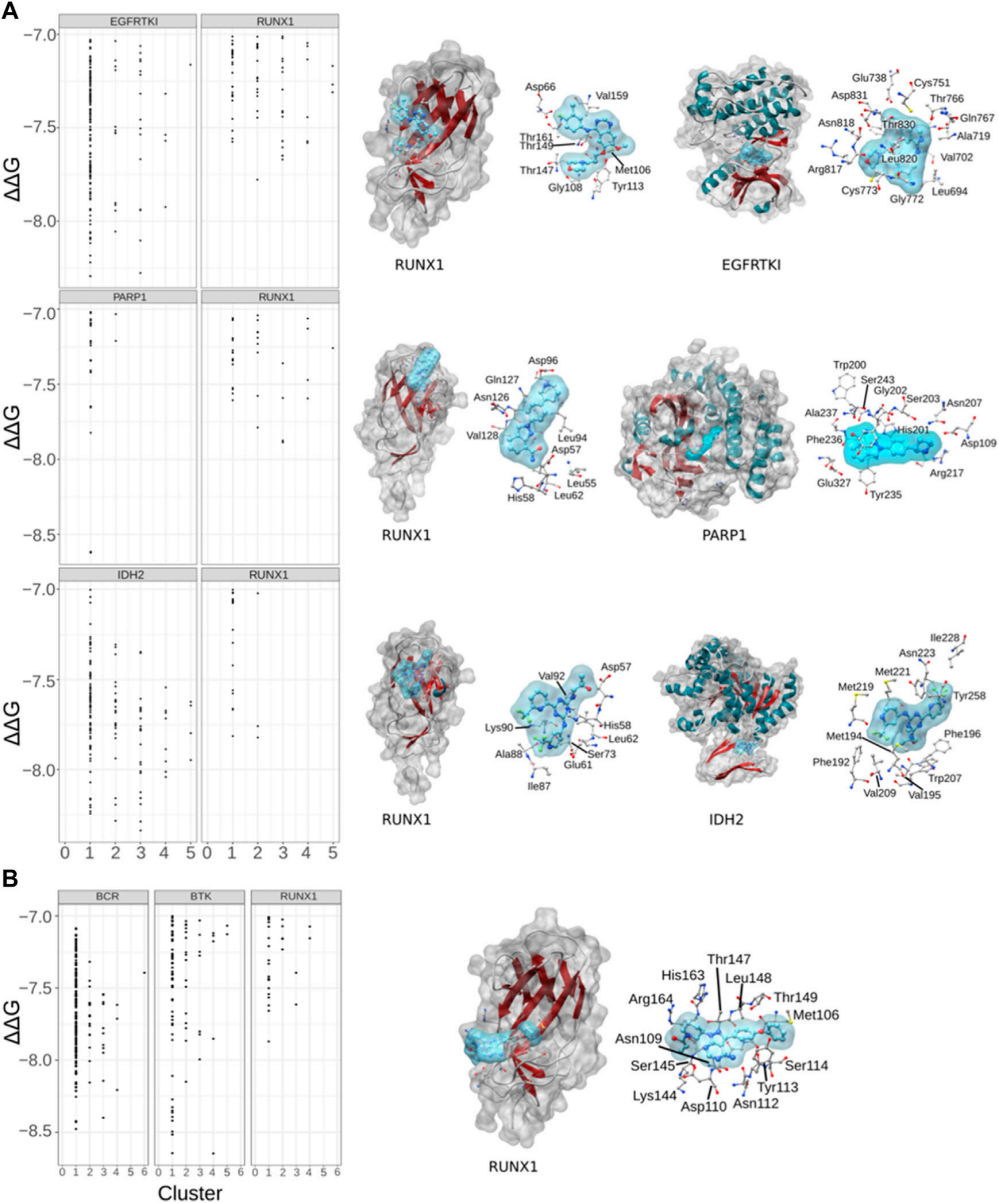
Drug associated targets and drug modeled binding with RUNX1. **(A)** Dot plots showing the comparative binding energy (∆∆G) for gefitinib (lung cancer), niraparib (ovarian cancer), and enasidenib (AML). Shown is the predicted gefitinib binding site versus the known binding area of gefitinib with its target protein EGFRTKI. Similarly, comparisons are shown with niraparib with its known protein target PARP1, and enasidinib and its established target IDH2. **(B)** Ibrutinib with its accepted targets BCR and BTK, versus that predicted with RUNX1.

We hypothesized that cancer-associated drugs would display higher modeled ΔΔG values for their expected/known targets and lesser ΔΔG values for RUNX1. On the basis of this prediction, we subjected Gefitinib, Niraparib, Ibrutinib, and Enasidenib for further docking studies with their respective published targets (Figure 4A). We performed docking of Gefitinib with its published target, the epidermal growth factor receptor tyrosine kinase (EGFRTKI, PDB ID: 3W2S). As expected, we found that Gefitinib exhibited more significant ΔΔG values for binding to EGFRTKI in comparison to RUNX1. RUNX1 residues Asp66, Met106, Gly108, Tyr113, Thr147, Thr149, Val159, and Thr161 were modeled to interact with Gefitinib (Figure 4A). Niraparib docking with Poly [ADP-ribose] polymerase 1Poly [ADP-ribose] polymerase 1 (PARP1) also showed a higher ΔΔG value for association with PARP1 than with RUNX1. RUNX1 amino acids Leu62, Asp57, His58, Leu62, Leu94, Asp96, Asn126, Gln127 and Val128 were modeled to interact with Niraparib (Figure 4A). Furthermore, we compared the docking of Enasidenib with its known target isocitrate-dehydrogenase-2 (IDH2), versus this drug’s docking with RUNX1. Similar to the other two comparisons, we found that the ΔΔG value is higher for the IDH2: Enasidenib complex than upon RUNX1: Enasidenib docking. RUNX1 amino acids Asp57, His58, Glu61, Leu62, Ser73, Ile87, Ala88, Lys90, and Val92 were modeled to interact with Enasidenib (Figure 4A). Ibrutinib showed a similar pattern of ΔΔG values when comparing the modeling of its binding to the B cell antigen receptor (BCR) relative to RUNX1. Ibrutinib is additionally known to inhibit Bruton’s tyrosine kinase (BTK). Our results suggested an equivalent ΔΔG value of Ibrutinib binding to the BCR receptor as to the BTK protein (Figure 4B). This result is consistent with Ibrutinib having more than one known target. Additionally, it leaves open the possibility of Ibrutinib binding to RUNX1 (pocket1). RUNX1 pocket1 residues Met106, Asn109, Asp110, Asn112, Tyr113, Ser114, Lys144, Ser145, Thr147, Leu148, Thr149, His163, and Arg164 suggested interactions with Ibrutinib. These residues are in proximity with cancer-associated mutations and are also located at the DNA binding interface of RUNX1 (Figure 4B). All-together, the docking results suggest that Gefitinib, Niraparib, and Enasidenib are more specific to their respective targets EGFRTKI, PARP1, and IDH2 in comparison to RUNX1. Here, we predicted that RUNX1 may be an effective target in Non-Hodgkin Lymphoma, and that the residues of RUNX1 that are involved in interacting with Ibrutinib are also in proximity to the DNA binding interface of RUNX1.

### Functional probability of cancer-associated mutations and their proximity with DNA

**FIGURE 5:**
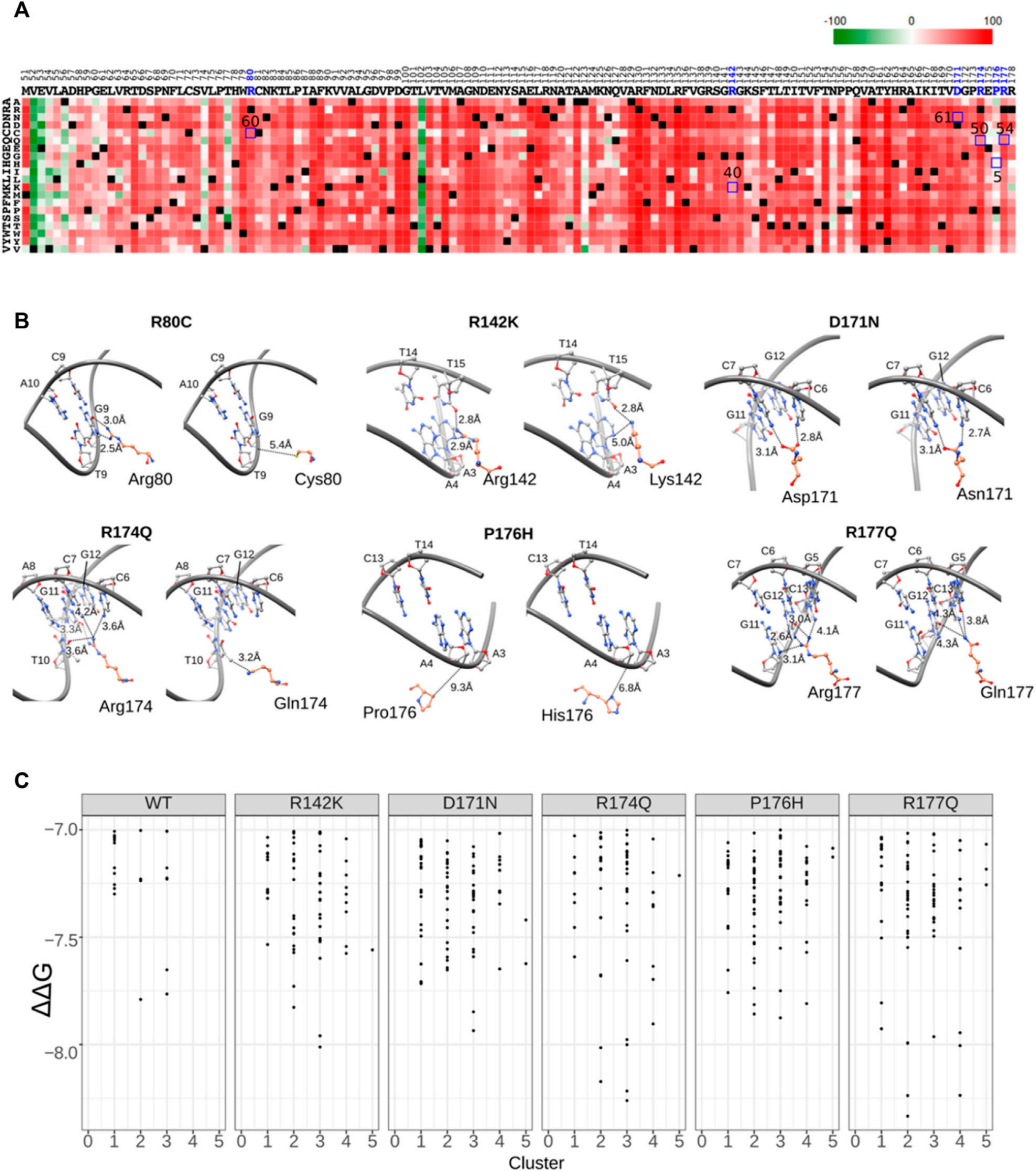
Mutation functional probability and DNA proximity.**(A)** Heatmap showing mutants’ probability of an impact upon functionality. Green color indicates lesser predicted impact while red color indicates higher impact. The *x* axis shows all amino acids of the RUNX1 DNA binding domain while the *y* axis shows the probability of mutation to each individual amino acid. Highlighted blue square boxes showing mutation probability scores for selected mutation represented in Figure 5B. **(B)** Cancer-associated missense mutation effect on molecular interactions, with the modeled and calculated distance between residues shown by dotted lines. DNA is shown as a gray ribbon and all residues are represented by ball and stick models. **(C)** Comparative docking of the AML associated drug molecule enasidenib with cancer-associated mutations of RUNX1 R142K, D171N, R174Q, P176H and R177Q versus wild type (WT).

Next, to evaluate the functional ability of RUNX1 mutants, we applied structural coordinates of RUNX1 in the context of the RUNX1-ETS TCR enhancer. We used the PredictProtein prediction tool to score for the impact of selected amino acid changes (Yachdav et al., 2014) (https://predictprotein.org/). The color green indicates a lower likelihood of functional changes, while the color red indicates a higher probability. The resulting scores suggest a high probability of functional effects for all but one of the evaluated residues (P176H) in RUNX1 that interact with DNA (Figure 5A). Thus, our results suggest that known mutations in RUNX1 that we predicted may affect its binding to DNA are likely to alter its function. To better evaluate these molecular interactions, we created a mutational model using UCSF Chimera 1.13v. We investigated if changes in DNA-binding likely occurred because of cancer-associated mutations in the proximity of the DNA-binding interface. The binding preferences for RUNX1 that harbor mutations, versus RUNX1 that harbor the corresponding normal residue were only slightly different in most cases. For example, when evaluating the predicted DNA-binding mutations Arg142Lys, Arg174Gln, and Arg177Gln, minor proximity differences were observed for the DNA-binding interface, with Pro176Gln showing no discernable difference relative to control/wild-type RUNX1. However, mutations in Arg80 and Cys80 predicted more significant differences in RUNX1 binding to DNA. Especially these latter predicted differences for the interaction of RUNX1 mutants with DNA could suggest RUNX1’s altered molecular function (Figure 5B). On the basis of these calculations to model molecular associations between RUNX1 and DNA, we added the consideration of the impact upon drug binding. In the context of the presence of the drug Enasidenib, we performed RUNX1: DNA-docking interactions employing the AML-associated RUNX1 mutants R142K, D171N, R174Q, P176H, R177Q in comparison to wild type (WT) RUNX1. Interestingly, we found that the docking scores of each of the RUNX1 mutants with Enasidenib was better than that with wild-type RUNX1, with R174Q and R177Q mutants having the highest ΔΔG values (Figure 5C), similar to that modeled for the docking of Enasidenib with its established target protein IDH2 (Sweta et al., 2019). Thus, we speculate that at least certain cancer-associated mutations in RUNX1B may exhibit the capacity to alter drug binding, and thereby facilitate drug binding to the RUNX1 protein.

## Discussion

Here, defined cancer-associated missense mutations of RUNX1 (R80C, R142K, D171, R174Q, P176H, and R177Q) are predicted to be relevant for DNA binding, and additionally in some cases, to drug binding. Mutation R80C (COSM24736) recurred 17 times and is found only in AML patients. Mutation R142K (COSM5028748) recurred 3 times and is associated with breast ductal carcinoma. Mutation D171N (COSM24721) recurred 26 times in AML patients (Supplementary Table S1). Mutation R174Q (COSM24721) recurred 33 times in AML patients. Indeed, amino acid position R174 is conserved across the paralogs and its recurrent mutation R174Q is significant for AML (Berardi et al., 1999; Song et al., 1999). Similarly, we predict that the mutations P176H (COSM5879709) and R177Q (COSM24731) are relevant to the DNA binding of RUNX1, with R177Q being highly recurring in AML patients. Although the specific molecular consequences of missense mutations are often unclear, here we attempt to address the impact of defined missense mutations in RUNX1 with its association with DNA. Additionally, we probe for effects upon the binding of drugs to RUNX1. Some drugs are used at lower levels when given in combination with other treatments because they would otherwise have unacceptable side effects, including those that are off target. Common drawbacks recognized from the application of more generalized drugs has aided the concept of personalized medicine. In common with many cancers, improved treatments for AML will benefit from better prognosis and available therapies (Lu et al., 2018). FDA-approved AML drugs such as Gilteritinib and Enasidenib are, respectively intended to target mutants of FLT3 or of IDH1/2 (Yang et al., 2019). Differences in drug responses has been attributed to corresponding differences in the classes of underlying driver mutations involved, that for example may encode epigenetic regulators, components of spliceosomes, or transcription factors such as IDH1, IDH2, SRSF2, U2AF, RUNX1, GATA2, and ETV6 (Ley et al., 2013; Metzeler et al., 2016; Papaemmanuil et al., 2016; Bullinger et al., 2017). AML is characterized by a genetically heterogeneous nature and a complex pattern of mutations which makes its treatment challenging (Tyner et al., 2018). Here, we combined molecular modeling and docking as tools to predict the molecular-interaction consequences of cancer-associated missense mutations of RUNX1 to binding DNA, as well as to binding defined drugs. We initially focused upon missense mutations predicted to be functionally relevant due to their involvement in mediating the DNA binding interface of RUNX1. For example, differences in drug binding efficacy could arise from the presence of such mutations. Interestingly our findings predict that the R174Q and R177Q mutations may facilitate the AML-associated drug Enasidenib interaction with RUNX1 (pocket1), and could provide additional deleterious effect on its DNA binding (Song et al., 1999). That is, these mutations may have combined effects, firstly at the DNA binding interface which appears consistent with the field’s current expectations, and conceivably also in regards to (off-target) drug binding, potentially producing presently unknown effects.

## Conclusion

In this study, we sought to identify RUNX1 mutants that may have a direct impact upon DNA association, and thus upon RUNX1 function. We also took 13 cancer-associated published drug compounds to model their possible binding to the transcription factor RUNX1. Here, we propose that structural molecular modeling and docking studies for RUNX1 in the presence of DNA, and/or drugs, may facilitate assessment of the potential impact of RUNX1 cancer associated mutations in AML. For example, mutation of R174Q and R177Q is predicted to compromise RUNX1:DNA binding interactions, while at the same time, facilitate off-target binding of the drug Enasidenib to RUNX1 (pocket1), with unknown consequences. Previously, this drug had not been proposed to associate with RUNX1. Enasidenib’s published target is IDH2, even though our modeling and docking suggest possible interactions (similar deltadeltaG scores) with the mutation R174Q or R177Q in RUNX1. As the field moves forward, understanding the molecular interaction consequences of RUNX1 mutations in AML will be important both in the context of DNA-binding as well as drug associations.

## Data Availability

The original contributions presented in the study are included in the article/Supplementary Material, further inquiries can be directed to the corresponding author.
