# Supplementary material for: In-silico probing of AML related RUNX1 cancer-associated missense mutations: Predicted relationships to DNA binding and drug interactions
Source: Front Mol Biosci. 2022 Aug 25;9:981020. doi: 10.3389/fmolb.2022.981020 (PMC9454315; doi:10.3389/fmolb.2022.981020)

Supplementary Figure 1: Workflow for mapping of mutations and docking of drugs

( ) = parentheses enclose the number of missense mutations.
DBD = DNA Binding Domain
PDB = Protein Data Bank
FATHMM = Functional Analysis Through use of the Hidden Markov Modell
FDA = Fedral Drug Administratsion.

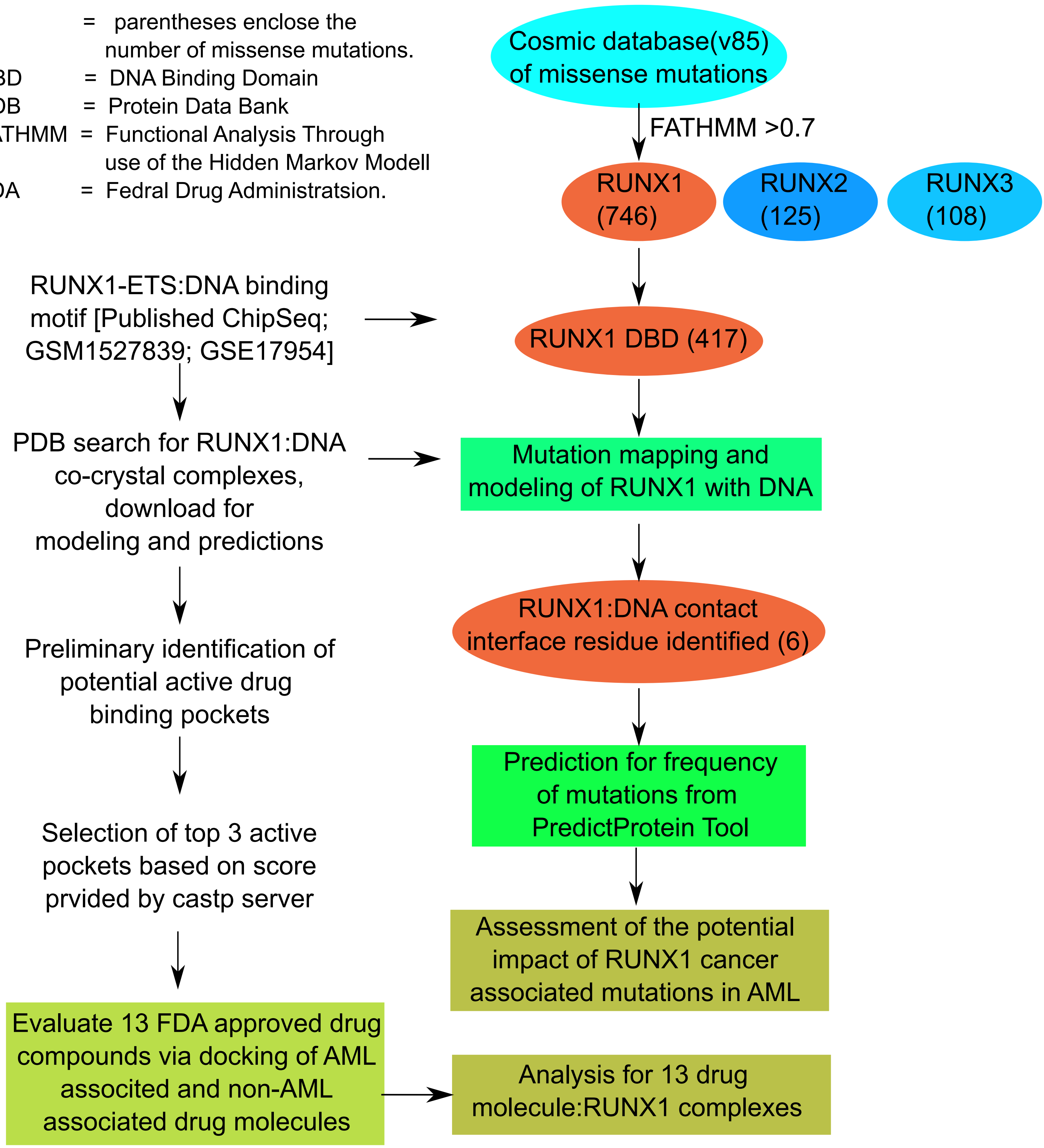

Supplement: Supplementary file 1 [file DataSheet1.PDF]
